# Dose-Dependent Effects of ZnO Nanoparticles Towards the Microalgae *Lobosphaera*: Compensation of Salt Stress at Low Concentration and Toxicity at High Concentrations

**DOI:** 10.3390/ijms26199455

**Published:** 2025-09-27

**Authors:** Olga V. Zakharova, Inna A. Vasyukova, Svetlana P. Chebotaryova, Elina Yu. Koiava, Svetlana S. Razlivalova, Grigory V. Grigoriev, Petr A. Baranchikov, Alexander A. Gusev

**Affiliations:** 1 Scientific and Educational Center for Environmental Science and Biotechnology, Derzhavin Tambov State University, 392000 Tambov, Russia; olgazakharova1@mail.ru (O.V.Z.); vasyukovaia@gmail.com (I.A.V.); sweta-chebotarjova@yandex.ru (S.P.C.); e.koiava.e.02@mail.ru (E.Y.K.); razlivalova8@yandex.ru (S.S.R.); bboykick@outlook.com (G.V.G.); petrovi4-98@yandex.ru (P.A.B.); 2 Department of Functional Nanosystems and High-Temperature Materials, National University of Science and Technology «MISIS», 119991 Moscow, Russia

**Keywords:** ZnO nanoparticles, microalgae, toxicity, salt stress compensation

## Abstract

This study investigated the concentration-dependent effects of zinc oxide nanoparticles (ZnO NPs, 30–70 nm) on the freshwater microalga *Lobosphaera* sp. under different salinity conditions (0–4 g L^−1^ NaCl). ZnO NPs demonstrated dual effects: low concentration (0.75 mg L^−1^) enhanced growth and alleviated salt stress, while higher concentrations (7.5–75 mg L^−1^) caused significant growth inhibition (up to 52%) and induced oxidative stress. Salinity did not significantly affect NPs aggregation patterns, and neither salinity nor aggregation degree influenced toxicity outcomes. NPs concentration plays a dominant role of toxicological effects. Dose-dependent increases in catalase activity and ROS-positive cells confirmed NPs-induced oxidative stress. Crucially, zinc bioaccumulation correlated with NPs concentration but dissociated from dissolved Zn^2+^ release, demonstrating particle-driven toxicity. Our findings challenge the ion-release paradigm and highlight the potential of low-dose ZnO nanoparticles as effective stress-protectors in algal biotechnology, offering new strategies for enhancing microalgal resilience under environmental stress.

## 1. Introduction

The importance of water resources, which occupy more than two-thirds of the Earth’s surface, cannot be overestimated. They are vital for the existence of all living things, and their deficit creates serious problems for the provision of the population and the agricultural sector [1]. Since water is the most important natural source, pollution of the aquatic ecosystem negatively affects both the environment and human well-being [2]. Domestic and industrial wastewater, runoff from agricultural land, etc., are the main sources of fresh water pollution [3]. The composition of such wastewaters may include chemicals, heavy metals, petroleum products, pesticides, antibiotics and hormones and a number of other dangerous pollutants [4].

Synthesized nanoparticles are a category of emerging pollutants that raise serious concerns due to their potential impact on marine communities and living organisms in coastal zones [5,6]. Zinc oxide nanoparticles (ZnO NPs) are ranked third in global production. This high figure is due to their use in numerous areas, from cosmetics and electronics to agriculture [7,8,9,10,11]. Potential negative impacts on aquatic organisms are associated with the large-scale release of these NPs into the environment, which is a consequence of their active use [12]. Direct measurements and computer modeling results indicate that concentration levels of ZnO NPs in the natural environment, according to various sources, range from 1.3 to 900 μg L^−1^ [13,14].

The LC50 value for ZnO NPs is less than 0.1 mg L^−1^, indicating their extreme toxicity to aquatic organisms [15]; however, toxicity to aquatic organisms belonging to different trophic levels varies considerably [7]. Among the species studied, invertebrates showed the greatest sensitivity to the impact, while vertebrates were the least sensitive. Bacteria and algae occupied an intermediate position [12]. The toxicity of ZnO NPs is primarily due to three factors: (1) the release of Zn^2+^ ions, (2) direct interaction of NPs with cells, and (3) the formation of reactive oxygen species (ROS) [16].

Despite extensive research into the impacts of heavy metals, pesticides, and NPs on freshwater environments [17,18,19,20,21,22], many other pollutants remain poorly understood [23]. Thus, anthropogenic impact, leading to changes in the salt composition and general mineralization of freshwater ecosystems [24], has been studied to a much lesser extent compared to other factors [23,25]. Two opposing processes are taking place: salinization of fresh waters as a result of human impact and dilution of naturally saline ecosystems by agricultural runoff. This occurs simultaneously with changes in other stressors that may have additive, antagonistic, or synergistic effects on organisms [26]. Decreased salinity increases the thermal sensitivity of marine and brackish organisms, as found in a meta-analysis of 90 studies of their thermotolerance (by parameters such as temperature optimum and limits). At the same time, several studies of freshwater species showed that their lower temperature limits increase and upper temperature limits decrease with increasing salinity, although the resulting figures were statistically insignificant [27]. Yang et al. have shown that phytohormones can more effectively stimulate microalgae growth and lipid content under salt stress [28]. Another study has shown an increase in the adsorption of cadmium and copper on the surface of diatom algae under the combined effect of silicon and water salinity [29]. Increasing water salinity shows a clear impact on the toxicity of silver NPs. Studies have shown that with increasing salinity from 35 to 140 g L^−1^, the median inhibitory concentrations of both Ag NPs and AgNO_3_ for *Dunaliella salina* (estimated by average specific growth rate and yield) increase significantly [30]. Similarly, another study found that at salinity up to 32 g L^−1^, larger agglomerates of NPs are formed, which reduces their toxic effect on algal cells [31]. Similar effects were obtained in [32], which also has shown an increase in the size of aggregates and a decrease in the release of ions with increasing salinity, which led to a decrease in the toxicity of ZnO NPs towards the marine diatom *Thalassiosira pseudonana*.

It is worth noting that most studies have focused on assessing the impact of NPs and salinity in relation to marine organisms [30,32,33,34]. At the same time, as stated earlier, salinization of freshwater bodies is a serious ecological problem, and the study of the influence of nanoscale pollutants under saline conditions is an important scientific task. Therefore, in our study we investigated the concentration effects of ZnO NPs on the unicellular freshwater microalgae *Lobosphaera* sp. against the background of different salinity of the medium. The important tasks here are to reveal the presence or absence of dose-dependent effects, as well as to determine the degree and nature of the influence of medium salinity and aggregation of NPs on their toxicological properties.

## 2. Results

### 2.1. Results of the Study of ZnO NPs and Their Suspensions

**Figure 1:**
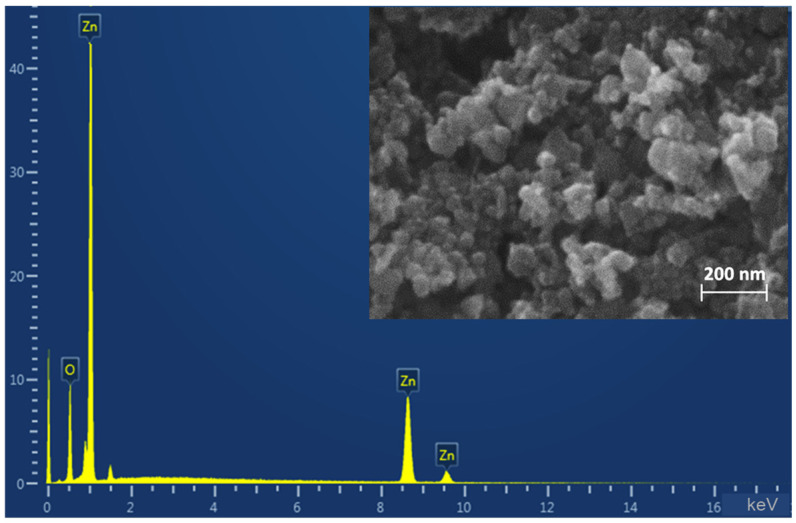
SEM-micrograph and energy dispersive X-ray spectroscopy.

By SEM method it was found that the composition of ZnO NPs powder includes both individual particles and their aggregates (Figure 1). Individual particles are in the size range of 30–70 nm, the shape of particles is close to spherical. The elemental analysis showed the presence of zinc and oxygen without any impurities in the sample composition.

**Figure 2:**
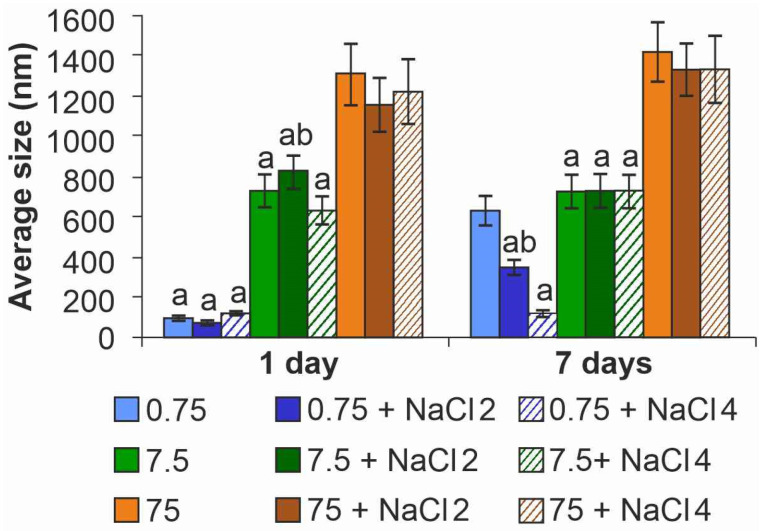
Analysis of variance of ZnO NPs. a—differences with higher NPs concentration, b—differences with higher salinity. Here and below NaCl 2 is the NaCl content in the medium 2 g L^−1^, NaCl 4 is 4 g L^−1^.

To study the behavior of ZnO NPs in dispersions with different salinity over time (1 and 7 days, the beginning and end of the experiment with microalgae), the size of particles and aggregates in solutions was analyzed with the DLS method. The results showed a significant effect of particle concentration on particle aggregation (Figure 2).

DLS analysis confirmed that NPs aggregation occurred in freshly prepared solutions (day 1) and was highly dependent on concentration. While the absolute sizes provided by DLS (ranging from 90 to 1200 nm) are hydrodynamic diameters that can be skewed by the intense light-scattering properties of large agglomerates, the key finding is the clear, concentration-dependent trend in aggregation. A nine-fold increase in the apparent aggregate size was observed when the NP concentration increased from 0.75 to 7.5 mg L^−1^. No significant influence of salinity on this initial aggregation was detected.

By the seventh day of the experiment, in the variant without sodium chloride and with a minimum content of NPs, a significant (almost eightfold) increase in particle size was noted—up to 628 nm compared to the size in the fresh suspension. The use of NaCl had a stabilizing effect: a dose of 2 g L^−1^ limited the growth of particles to 350 nm, and 4 g L^−1^ completely prevented aggregation, maintaining the diameter at the level of the first day (~100 nm). It is worth noting that this effect of NaCl was only observed for dispersions with a concentration of NPs of 0.75 mg L^−1^. For the suspensions containing 7.5 and 75 mg L^−1^ the parameters did not differ from the parameters on the first day of the experiment.

The results of the evaluation of the release of zinc ions into the cultivation medium showed the absence of the influence of the NPs concentration on this indicator. All measured values were in the range of < 0.1 mg L^−1^, at the level of the background zinc content (0.05 mg L^−1^) in the BG 11 nutrient medium (Supplementary materials, Table S1).

### 2.2. ZnO NPs Effect upon Lobosphaera sp.

#### 2.2.1. Analysis of Total Cell Numbers and Cell Viability

**Figure 3:**
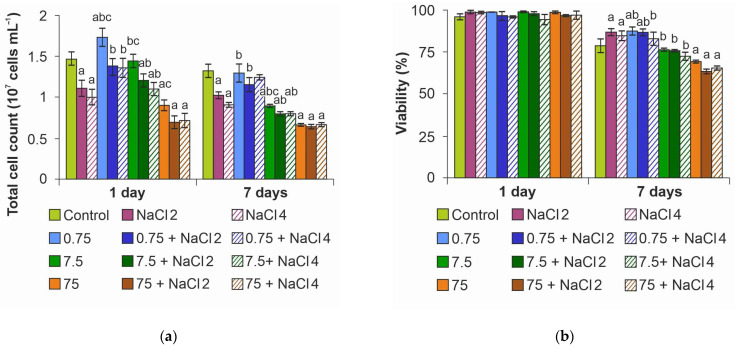
Effect of ZnO NPs on *Lobosphaera*: (**a**) total cell number; (**b**) viability. a—differences with control, b—differences with higher concentration of ZnO NPs, c—differences with higher salinity.

Analysis of the total cell number showed a decrease in the index with increasing salinity of the medium as early as on day 1 of the experiment (Figure 3a). A concentration of 0.75 mg L^−1^ ZnO NPs increased cell numbers by 19%, whereas NaCl neutralized this effect, returning cell numbers to control levels. When NPs concentration was increased to 7.5 mg L^−1^ in medium with NaCl, cell number decreased by 21%, while 75 mg L^−1^ NPs decreased the index by 38% in medium without salt and by 52% upon salinization of the medium regardless of NaCl concentration. A similar trend was maintained on day 7 of the experiment; however, an even greater decrease in cell number was observed in the groups with 7.5 mg L^−1^ and 75 mg L^−1^ concentrations. In general, it can be noted from the experiment that NaCl concentration mattered in the medium without NPs, while in dispersions with NPs the dose-dependent effects of NaCl disappeared.

Evaluation of microalgae culture viability under the action of ZnO NPs and NaCl showed that, despite the decrease in the number of cells, on the first day of the experiment, the cell viability indices were at the level of control cells (Figure 3b). It is noteworthy that on day 7 of the experiment, no decrease in the viability of cultures under the action of NaCl as well as ZnO NPs at concentrations of 0.75 and 7.5 mg L^−1^ was detected. Moreover, at salinization of the medium an increase in viability by an average of 8% was observed simultaneously with a decrease in the number of cells. The decrease in the index by 10–15% was recorded only at the maximum concentration of NPs.

#### 2.2.2. Analysis of Pigment Content and Photosynthesis Activity

**Figure 4:**
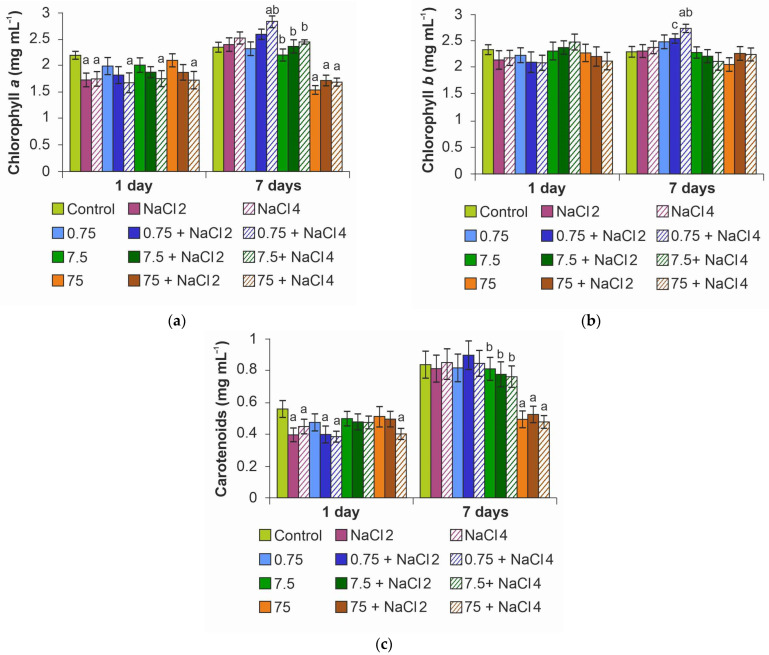
Effect of ZnO NPs on pigment content in *Lobosphaera* cells: (**a**) chlorophyll *a*; (**b**) chlorophyll *b*; (**c**) carotenoids. a—differences with control, b—differences with higher concentration of ZnO NPs, c—differences with higher salinity.

NaCl application decreased chlorophyll *a* content in microalgae cells on day 1 of exposure regardless of NPs addition (Figure 4a). At the same time, ZnO NPs had no significant effect on the index. At the end of the experiment, the negative effect of salinity disappeared; moreover, when algae were cultured in a medium with 0.75 mg L^−1^ ZnO NPs and 4 g L^−1^ NaCl, the content of chlorophyll *a* increased by 17%. A rise in the concentration of NPs to 75 mg L^−1^ had a suppressive effect on pigment synthesis, the concentration of which decreased by 32% on average.

In the case of chlorophyll *b*, there was no effect of ZnO NPs and NaCl on its content, except for an 18% increase on day 7 of the experiment in the 0.75 mg L^−1^ ZnO NPs + 4 g L^−1^ NaCl group (Figure 4b).

Carotenoid content, as well as chlorophyll *a*, generally decreased under NaCl on the first day of the experiment (Figure 4c). However, on day 7, the decrease in carotenoid concentration was recorded only at the maximum dose of ZnO NPs, irrespective of the medium salinity.

**Figure 5:**
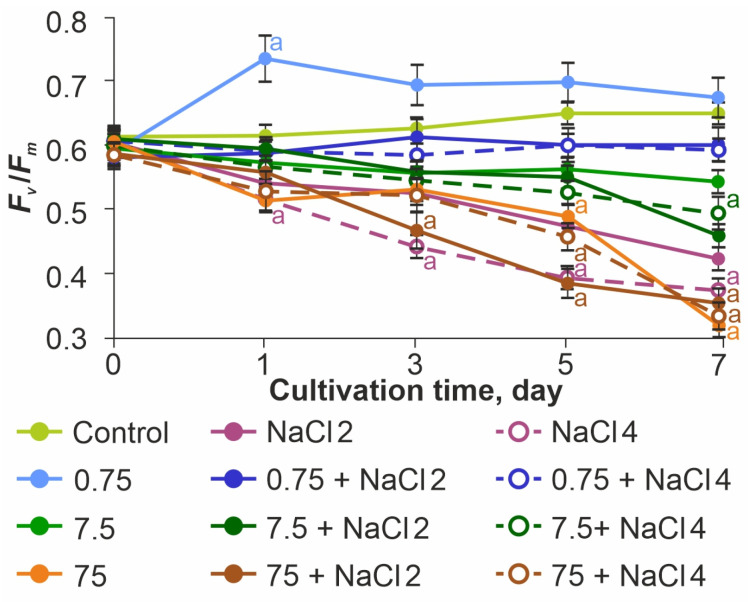
Effect of ZnO NPs on the photosynthetic efficiency of *Lobosphaera* sp. The letter denotes the presence of significant differences with the control.

To indicate the influence of the studied stress factors on photosynthetic activity, the *F_v_*/*F_m_* index was used, which characterizes the functional resistance of the photosynthetic apparatus to external influences (Figure 5).

In the control sample, this parameter maintained the optimal value of 0.61–0.65 throughout the experiment. Before the beginning of exposure, the value of *F_v_*/*F_m_* was similar to the control sample in all experimental samples. When NaCl was added at concentrations of 2 and 4 g L^−1^, a significant decrease in photosynthetic activity was noted throughout the observation period. On the last day of the experiment the *F_v_*/*F_m_* values were reduced relative to the control by 33.3% at 2 g L^−1^ NaCl and by 43.6% at 4 g L^−1^ NaCl, confirming the negative effect of increased medium salinity for the test subject.

ZnO NPs at a concentration of 0.75 mg L^−1^ had a stimulatory effect on PSII, with *F_v_*/*F_m_* reaching values of 0.73–0.75 on the first day. At the same time, no such effect was observed in ZnO NPs 0.75 mg L^−1^ + 2 g L^−1^ NaCl and ZnO NPs 0.75 mg L^−1^ + 4 g L^−1^ NaCl samples. In these groups, *F_v_*/*F_m_* values were almost equal to the control values (less than 9% difference from control).

In the samples of ZnO NPs 7.5 mg L^−1^ and ZnO 75 mg L^−1^, regardless of the presence of NaCl, a decrease in photosynthetic activity was observed throughout the experiment. The minimum values of photosynthetic activity were observed in the ZnO NPs 75 mg L^−1^ + 2 g L^−1^ NaCl group.

#### 2.2.3. Oxidative Stress Analysis

**Figure 6:**
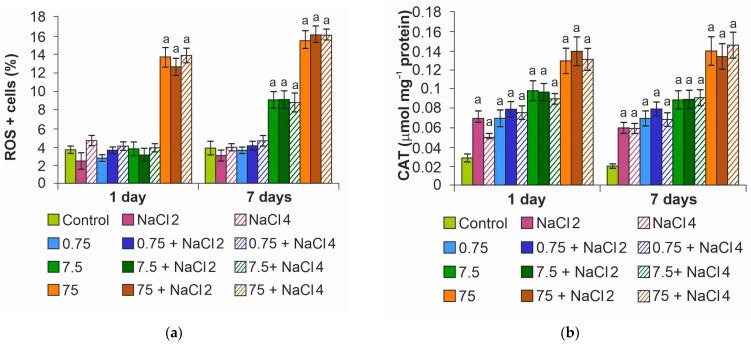
Oxidative stress analysis: (**a**) quantification of the percentage of ROS + cells; (**b**) catalase activity. The letter a denotes the presence of significant differences with the control.

Quantitative determination of cells with ROS showed the appearance of cells with oxidative stress already on the first day of the experiment at the maximum concentration (Figure 6a). At the end of the experiment, the percentage of cells with oxidative stress increased proportionally to the concentration of NPs, reaching a maximum at 75 mg L^−1^. At the minimum dose of ZnO NPs, the indicators were close to the control ones.

Catalase activity increased in the variants with NPs (Figure 6b), starting from the minimum concentration of ZnO NPs. It is likely due to the activation of the antioxidant system at 0.75 mg L^−1^ NPs that oxidative stress was not observed in algae cells. It is worth noting that for cells exposed to a medium with NaCl without NPs, a slight increase in enzyme activity was also noted.

#### 2.2.4. Protein Content

**Figure 7:**
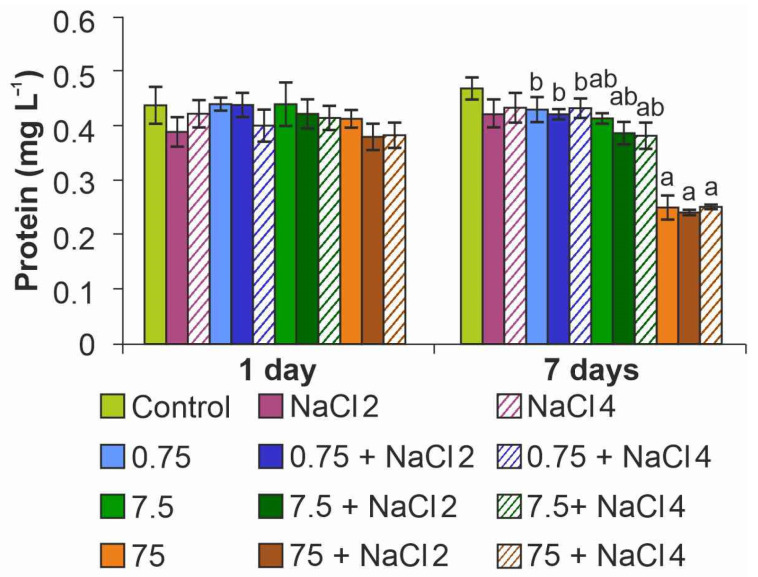
Effect of ZnO NPs on protein content in *Lobosphaera* cells. a—differences with control, b—differences with higher concentration of ZnO NPs.

Analysis of protein content in microalgae cells showed a significant decrease in the index on day 7 at maximum NPs concentration, on average by 50%, regardless of medium salinity (Figure 7). When algae were cultured in medium with 7.5 mg L^−1^ ZnO NPs, the protein concentration decreased by 20%. The stimulating effect of ZnO NPs on protein synthesis in cells was not established in this experiment.

The analysis of the results shows that under the joint action of two factors—ZnO NPs and NaCl, the concentration of ZnO NPs is the key parameter affecting the microalgae. According to the results, the minimum of the used NPs concentrations either had no effect or stimulated the growth of the indicators. In addition, in some cases the addition of 0.75 mg L^−1^ ZnO NPs to the medium with NaCl leveled out the negative effect of salinization of the medium. The concentration of NPs of 7.5 mg L^−1^ generally had no effect on the analyzed parameters, but it also did not reduce the negative effect of NaCl. At the maximum concentration of NPs toxic effects were detected in most cases, the result had little dependence on NaCl concentration.

#### 2.2.5. Zn Content

Analysis of Zn content in microalgae also showed the crucial role of NPs concentration in metal accumulation in cells. The maximum metal content (3.03 ± 0.04 pg cell^−1^) was observed in cells cultured at 75 mg L^−1^ NPs, regardless of medium salinity. At the concentration of 7.5 mg L^−1^ the Zn content was 0.38 ± 0.01 pg cell^−1^. At the lowest concentration of NPs Zn accumulation in cells was not recorded.

**Figure 8:**
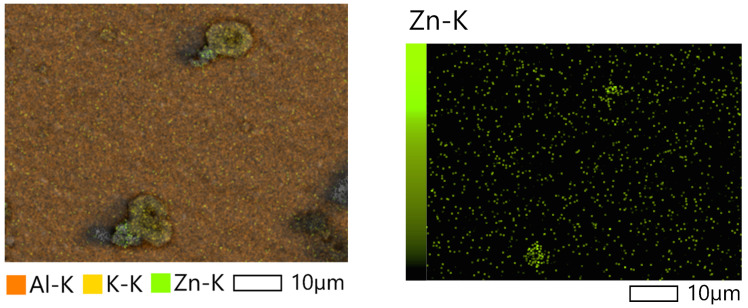
SEM- images of *Lobosphaera* cells cultivated with 75 mg L^−1^ ZnO NPs.

Electron microscopic study with elemental mapping showed accumulation of ZnO particles on the cell surface (Figure 8).

The results of the study suggest that ZnO NPs most likely do not penetrate into cells but are sorbed on their surface.

## 3. Discussion

Our results show the key influence of the concentration of ZnO NPs on their aggregation in the dispersion medium. In our study the size of aggregates increased linearly with increasing concentration. It is known that the concentration of particles determines the distance between particles and is an important parameter for their stability [35]. Decreasing the concentration of NPs can lead to enhanced dissolution and decreased aggregate size [36]. At the same time, for freshly prepared dispersions, no effect of the medium salinity on aggregation was detected, whereas on the 7th day of the experiment at the minimum dose of ZnO NPs, the particle size decreased from 630 nm to 108 nm with increasing salinity. Overall, the aggregation pattern of NPs did not have the expected effect on toxicity (normally, the toxicity of NPs increases with decreasing particle size due to a higher surface activity [37]).

When studying the effect of NaCl on microalgae, a decrease in cell number was observed when the medium was salinized. One of the leading limiting factors for the growth and development of microalgae is salt stress, which negatively affects the associated biochemical and physiological mechanisms [38]. High salinity level causes ionic, osmotic, and oxidative stress [39]. NaCl damages the oxygen-evolving complex and the reaction center of PSII, which inhibits electron transport on its donor and receptor sides [40].

At the same time, a significant number of studies show that microalgae cells can develop a metabolic response to salt stress, which consists of the accumulation of key molecules involved in membrane remodeling and stress resistance [38,41].

In our study, we observed an increase in the activity of the enzyme catalase in response to salinization of the environment. This is probably part of the adaptive response of the antioxidant defense system to cope with oxidative stress caused by high salinity [42].

ZnO NPs at the lowest concentration (0.75 mg L^−1^) had no adverse effect on the studied algae, they also offset the negative effects of salinity. The mechanism by which exogenous zinc alleviates the effects of abiotic stress is related to its positive effect on the main photosynthetic characteristics. These include net photosynthetic productivity, photosynthetic pigment concentration, chlorophyll fluorescence parameters, photochemical quantum efficiency, Rubisco enzyme activity, and electron transport in thylakoid membranes [43,44,45]. Another important function of zinc is the regulation of key metabolic pathways, including protein synthesis, photosynthesis processes, auxin formation, and lipid metabolism [43]. Zinc is a cofactor for many antioxidant defense enzymes, including superoxide dismutase, which are responsible for the detoxification of ROS [46]. For example, the study performed on *Phaseolus vulgaris* L. culture has shown a decrease in salt stress under the action of ZnO NPs [47]. The authors found that ZnO NPs promote accelerated plant growth and reduce ROS formation, thus regulating the homeostasis of nutrients in plants and chlorophyll fluorescence activity. In our study, we also noted the activation of the antioxidant system in the presence of ZnO NPs, as evidenced by the increase in catalase activity.

The increase in the content of NPs in the environment has gradually changed their role from stress protector to toxicant. ZnO NPs (7.5 mg L^−1^) had no effect on the culture at the beginning of the experiment and decreased the number of cells on day 7, while NaCl increased the inhibitory effect. At the same time, cell viability and photosynthesis activity indices were at the level of control values. However, at the maximum concentration (75 mg L^−1^) of NPs, the growth suppression of microalgae culture was observed as early as on day 1 and the effect increased under the influence of NaCl. At the end of the experiment, the index was almost twice below the control values regardless of NaCl application. Also, in groups cultured with 75 mg L^−1^ ZnO NPs, a decrease in cell viability, as well as a decrease in the concentration of chlorophyll *a* and carotenoids was recorded. Protein content in cells decreased on the 7th day of the experiment at 7.5 mg L^−1^ ZnO NPs (by 20%), as well as in all the groups with the maximum dose of NPs where an average decrease of 50% was noted. The absence of NaCl’s effect on ZnO NPs toxicity on day 7 is probably due to cell adaptation to salt stress. This version is supported by a slight increase in the viability of *Lobosphaera* sp. cells at the end of the experiment when cultured with 2 and 4 g L^−1^ NaCl, as well as the leveling of the negative effect on the photosynthetic system and protein synthesis. According to the literature [48], salt stress causes a complex set of reactions designed to protect the algal cell from osmotic stress. These reactions include a decrease in membrane permeability to protect the cell, activation of energy-consuming processes for the absorption of potassium ions and the removal of excess sodium ions, the synthesis of organic osmolytes that equalize osmotic pressure safely for the cell, and changes in the expression of genes that control the synthesis of antioxidants and membrane lipids to adapt to changed osmotic conditions. Thus, microalgae cells can experience an increase in salinity several times compared to the norm.

ZnO NPs at a concentration of 0.75 mg L^−1^ had a stimulatory effect on PSII. This is probably what led to a significant increase in the number of cells in culture (Figure 3a). However, no such effect was observed in the ZnO NPs 0.75 mg L^−1^ + 2 g L^−1^ NaCl and ZnO NPs 0.75 mg L^−1^ + 4 g L^−1^ NaCl samples. In these groups, the *F_v_*/*F_m_* values were maintained at the level of control values. There was likely a synergetic effect of photosynthetic apparatus stimulation by the action of ZnO NPs at 0.75 mg L^−1^ and compensatory reaction of the photosystem during adaptation to the medium salinization.

In ZnO NPs 7.5 mg L^−1^ and ZnO NPs 75 mg L^−1^ samples, regardless of the presence of NaCl, a decrease in photosynthetic activity was observed throughout the experiment. A decrease in *F_v_*/*F_m_* together with the change in the indices of photosynthetic pigments content in cells led to a slowdown in the growth rate of microalgae culture, which caused a decrease in the number of cells. As a result, we can speak about the general toxic effect of ZnO NPs in concentrations of 7.5–75 mg L^−1^. The reduction in this index under the action of ZnO NPs is also observed in the works of other authors [49]. The main mechanism of toxicity is most likely oxidative stress caused by the adsorption of ZnO NPs on the cell surface, which is supported by the detected increase in zinc content in the algal biomass, especially at the maximum concentration. At the same time, the large size of the aggregates (more than 1 μm) makes it unlikely that NPs will penetrate into cells. It is worth noting that the widespread version of the key influence of metal ions and their penetration into cells was not confirmed in our study—measuring the content of Zn^2+^ ions in the medium showed the absence of a significant difference between the experimental variants. A number of works by other authors also showed the main role in the toxicity of NPs, and not dissolved ions [50,51].

Thus, our results deviate from the conventional view that salinity and aggregation state are key modulators of ZnO NPs toxicity. The finding that neither factor significantly altered the toxic outcome points to the concentration of ZnO NPs themselves as the dominant predictor of biological effects. The beneficial role at low concentration (0.75 mg L^−1^), evidenced by growth stimulation and catalase induction, is consistent with a protective activation of the algal antioxidant system. In contrast, the toxicity at high concentrations (7.5–75 mg L^−1^), confirmed by oxidative stress markers and photosystem damage, indicates a breakdown of these defense mechanisms. This clear concentration-dependent duality, from protector to toxicant, underscores that the biological activity is an inherent property of the nanoparticulate form. Our data therefore emphasize that for predicting the environmental impact of ZnO NPs, the exposure concentration is a more reliable parameter than forecasts based on salinity or aggregation behavior.

## 4. Materials and Methods

### 4.1. ZnO NPs and Their Suspensions

ZnO NPs manufactured by Sigma-Aldrich (St. Louis, MO, USA) with a reported size < 50 nm were used in this work. Scanning electron microscopy (SEM; Tescan Vega 3 microscope, TESCAN GROUP, a.s., Brno, Czech Republic) was used to analyze the particle morphology. The elemental composition was determined by energy-dispersive X-ray spectroscopy (EDS) using an X-Act 10 mm^2^ SDD detector (Oxford Instruments, Oxford, UK).

Concentrated dispersions of ZnO NPs were prepared for introduction into the nutrient medium. Weighed portions of NPs powder (4.5, 45, 450 mg) on an Ohaus analytical balance (Ohaus Corporation, Parsippany, NJ, USA) were added to 100 mL flasks containing sterile distilled water (pH 7.1 ± 0.2), mixed and subjected to ultrasonic treatment for 10 min (ultrasonic bath VBS-41H, 180 W, 4 l, Vilitek, Moscow, Russia). Particle powders (4.5, 45, and 450 mg).

### 4.2. Cell Culture and Culture Conditions

The study was conducted on a culture of microalgae *Lobosphaera* sp. IPPAS C-2047 (collection of the K.A. Timiryazev Institute of Physiological Research, Russian Academy of Science), which was cultivated on BG-11 medium [52,53] in 250 mL glass conical flasks. Cultivation conditions included constant illumination with white LED lamps (480 μE/(m^2^ s)), temperature of 25 ± 1 °C, pH 7.1 ± 0.2 and constant shaking on an orbital shaker (Stegler, Shanghai, China).

### 4.3. Experiment Design

A total of 6 mL of BG-11 culture medium containing microalgae culture of *Lobosphaera* sp. (OD_678_—0.5) was added to the wells of a 6-well plate. Then, 100 µL of ZnO NPs suspension was added to each well and mixed. For control, 100 µL of sterile distilled water was added to the wells. The final concentrations of ZnO NPs for the exposure of *Lobosphaera* sp. were 0.75, 7.5, and 75 mg L^−1^ nutrient medium. The NPs concentrations were chosen taking into account the results of a preliminary study (Supplementary materials, Figure S1), where we found that the used ZnO NPs had no effect on *Lobosphaera* sp. at concentrations below 0.75 mg L^−1^.

To study the effect of medium salinity on the toxic effect of ZnO NPs, BG-11 culture medium containing 2 and 4 g L^−1^ NaCl was prepared. The experiment was laid out similarly to the experiment without NaCl.

The behavior of ZnO NPs in BG-11 and BG-11 + NaCl media was analyzed by measuring the average particle size on a Zetasizer Nano ZS analyzer (Malvern Instruments, Malvern, UK) on days 1 and 7.

To assess the effect of zinc ions on microalgae, measurements of Zn^2+^ concentrations were carried out for dispersions using the liquid analyzer “Expert-001” (Econix Expert, Moscow, Russia).

### 4.4. Cell Indicators

#### 4.4.1. Cell Numbers and Cell Viability

Total cell numbers in the presence of ZnO NPs and in control samples were assessed on day 1 (after 3 h of exposure) and day 7 of the experiment using a Muse Cell Analyzer, (Merck Millipore, Darmstadt, Germany).

Viability was also determined on days 1 and 7 using propidium iodide [54] by means of a Muse Cell Analyzer, (Merck Millipore, Darmstadt, Germany). The aliquots were resuspended in phosphate-salt buffer (PBS; pH 7.4) before analysis.

#### 4.4.2. Analysis of Pigment Content and Assessment of Photosynthetic Activity

The pigment composition of microalgae was quantitatively determined using extraction with dimethyl sulfoxide (DMSO, Sigma-Aldrich, St. Louis, MO, USA). The extraction protocol included the following steps: centrifugation of 1 mL of cell suspension (5 min, 6000 rpm, MiniSpin centrifuge, Eppendorf, Hamburg, Germany), removal of the supernatant, subsequent incubation of the cell pellet in DMSO at 70 °C for 10 min with vigorous mixing, and final sedimentation of the cells by centrifugation.

Spectrophotometric measurement of the concentrations of chlorophyll *a* (Chl-a), chlorophyll *b* (Chl-b), and the sum of carotenoids in the extract was performed on a Multiskan Sky microplate spectrophotometer (Thermo Scientific, Waltham, MA, USA) using 96-well plates. The calculation was performed using Formulas (1)–(3) [55]:

Chl-a = 13.34 D_666_ − 4.85 D_650_, (1)

Chl-b = 24.58 D_650_ − 6.65 D_666_, (2)

CR = (1000 D_480_ − 1.29 Chl-a − 53.76 Chl-b)/220, (3)

where Chl-a, Chl-b, CR—concentrations of chlorophyll *a*, chlorophyll *b*, and total carotenoids in the extract, respectively, mg L^−1^; D_λ_—optical density value measured at wavelength λ, nm.

To estimate photosynthetic activity of microalgae *Lobosphaera* sp., *F_v_*/*F_m_ =* (*F_m_* − *F*_0_)/*F_m_* i.e., the maximum potential photochemical quantum yield of photosystem II was registered, where *F*_0_ and *F_m_* are the minimum (when all reaction centers of photosystem II are open) and maximum (when all reaction centers of photosystem II are closed) levels of chlorophyll fluorescence, respectively [56].

Registration was performed using a PAR-FluorPen FP 110 fluorimeter (Photon Systems Instruments, Drasov, Czech Republic) on days 0, 1, 3, 5, and 7 of the experiment. Before measurement, the algae samples were kept in the dark for 15 min.

#### 4.4.3. Oxidative Stress Determination

The degree of oxidative stress was also carried out using the Muse Cell Analyzer (Merck Millipore, Darmstadt, Germany) and a Muse Oxidative Stress kit (Luminex Corporation, Austin, TX, USA). The kit allows for quantitative measurement of the activity of oxygen radicals (ROS) in cells with oxidative stress. The protocol allows for rapid statistically reliable results on the percentage ratio and concentration of cells: ROS- (living cells) and ROS+ (cells with active forms of oxygen). The activity of catalase, an antioxidant enzyme that breaks down hydrogen peroxide into water and oxygen, was also determined. Catalase plays a vital role in the protective systems of microalgae, protecting cells from oxidative stress caused by various environmental factors. The enzyme activity was determined spectrophotometrically on a Multiskan Sky device (Thermo Scientific, Waltham, MA, USA), according to the method in [57].

#### 4.4.4. Protein Content Analysis

The content of alkaline-soluble proteins in the microalgae culture was determined by the standard Bradford method [58,59]. First, 50 mL of experimental and control samples were centrifuged at 3500 rpm for 15 min. The supernatant was removed and the resulting microalgae precipitate was dried naturally. Then, 0.1 M NaOH was added to the samples and the obtained suspensions were processed in an Elmasonic S30H ultrasonic bath (ultrasonic power—80 WT, volume—2.75 L, Elma, Singen, Germany) for 20 min. Homogenates were centrifuged at 4000 rpm for 15 min. Protein concentration in the extract was determined using Coomassie Brilliant blue G-250 dye (Sigma-Aldrich, St. Louis, MO, USA) according to the Bradford method at 595 nm on a Multiskan Sky spectrophotometer (Thermo Scientific, Waltham, MA, USA). The calibration graph was plotted against bovine serum albumin (Proliant Biologicals, Ankeny, IA, USA).

#### 4.4.5. Zn Accumulation Analysis in Microalgae

Zinc content in microalgae biomass was quantitatively determined using inductively coupled plasma atomic emission spectrometry (ICP-AES) on a Varian 720-ES instrument (Agilent Technologies, Santa Clara, CA, USA). Cultures treated with NPs at maximum concentrations were selected for analysis. An aliquot of the suspension with a volume of 2 mL was filtered through Millipore membrane filters (pore size 0.45 μm, Bedford, MA, USA). Further sample preparation for ICP-AES analysis was carried out in accordance with the standard procedure [60].

In addition, to visualize the distribution of zinc, an electron microscopic study of microalgae cells cultivated at the maximum concentration of ZnO NPs was carried out. The work was carried out on a scanning electron microscope JCM-7000 NeoScope (JEOL, Tokyo, Japan).

### 4.5. Statistical Analysis

To ensure the reliability of the results, all experiments were carried out in three independent biological replicates, each of which included up to five technical replicates (their number varied depending on the analysis method). Statistical significance of differences from the control group (without pollutant) at the level of *p* < 0.05 was determined using one-way ANOVA. In the graphs and tables, data are presented as mean ± standard deviation; asterisks denote statistically significant differences.

## 5. Conclusions

In conclusion, our study challenges the established view that Zn^2+^ ion release is the primary driver of ZnO NPs toxicity by demonstrating that their toxicity towards the freshwater microalga *Lobosphaera* sp. under salt stress is not primarily driven by the release of dissolved Zn^2+^ ions. Contrary to numerous previous reports [16,61,62], our analysis revealed that the concentration of bioavailable ions was insufficient to account for the observed high toxicity at elevated NP concentrations. Thus, we successfully determined that the nature of the ZnO NPs’ influence is primarily particle-specific, rather than being mediated by salinity-dependent ionic release or aggregate size.

The most original and significant finding of this work is the identification of a pronounced particle-specific effect. The dose-dependent toxicity strongly correlates with the presence and concentration of the nanoparticulate form itself. We propose that direct interactions between ZnO NP aggregates and algal cells (e.g., surface catalysis, physical disruption, and particle-induced ROS generation), confirmed by a dose-dependent increase in oxidative stress markers and catalase activity, are the dominant mechanisms, rather than ionic dissolution. This insight is crucial for accurately predicting the environmental risk of ZnO NPs, as it shifts the focus from ion-regulated models to more complex particle-specific interactions.

This finding contrasts with several studies on marine diatoms [32] and other models where salinity mitigated toxicity by influencing aggregation and ion release. In our case, for the freshwater *Lobosphaera* sp., the minimal influence of salinity on toxicity further supports the conclusion that classical ion-mediated mechanisms are not the primary pathway.

Furthermore, the novel compensatory effect observed at low ZnO NP concentrations (0.75 mg L^−1^)—where NPs enhanced growth and mitigated salt stress—also appears to be a particle-specific phenomenon. We hypothesize that at low doses, NPs may act as mild stressors that induce a protective antioxidant response (e.g., increased catalase activity), thereby “priming” the algal defense systems and enhancing their resilience to subsequent salt stress, a mechanism that warrants further molecular investigation.

A limitation of this study is the absence of experimental data with soluble zinc salts, which would allow for a more precise quantification of the ionic contribution to the overall toxicity. Future research should include such controls to fully decouple the particle-specific effects from the indirect effects mediated by Zn^2+^ ion release.

In summary, our study provides compelling evidence that the environmental impact of ZnO NPs on freshwater microalgae is a function of concentration-dependent, particle-specific toxicity rather than solely dissolved ion release and is surprisingly independent of salinity stress and aggregation dynamics. This underscores the critical importance of the particulate nature of nanomaterials in assessing their ecotoxicological profile. The practical implications of our work are substantial: environmental risk assessment models for NPs must be revised to account for these particle-driven effects, especially in multi-stressor scenarios like freshwater salinization. Future research should focus on elucidating the exact mechanisms of this particle-cell interaction, including the role of aggregate size, surface chemistry, the specific pathways of oxidative stress induction, and the resulting oxidative stress pathways.

## Data Availability

Data are contained within the article.

## Supplementary Materials

The following supporting information can be downloaded at: https://www.mdpi.com/article/10.3390/ijms26199455/s1.
