# Supplementary material for: Dose-Dependent Effects of ZnO Nanoparticles Towards the Microalgae Lobosphaera: Compensation of Salt Stress at Low Concentration and Toxicity at High Concentrations
Source: Int J Mol Sci. 2025 Sep 27;26(19):9455. doi: 10.3390/ijms26199455 (PMC12525374; doi:10.3390/ijms26199455)
Supplement: Supplementary file 1 [file ijms-26-09455-s001.zip › ijms-3837970-supplementary.pdf]

International Journal of *Molecular Sciences*

MDPI

Supplementary materials

Dose-Dependent Effects of ZnO Nanoparticles Towards the Microalgae *Lobosphaera*: Compensation of Salt Stress at Low Concentration and Toxicity at High Concentrations

Table S1. Zn^{2+} concentration measurements results, mg L^{-1}.

Variants	Days after preparation						
	1	2	3	4	5	6	7
BG-11	0.05±0.002	0.04±0.009	0.05±0.002	0.05±0.002	0.04±0.008	0.05±0.002	0.05±0.002
BG-11 + 0.75 mg L^{-1} ZnO NPs	0.05±0.001	0.04±0.009	0.04±0.008	0.05±0.002	0.04±0.008	0.06±0.006	0.05±0.005
BG-11 + 7.5 mg L^{-1} ZnO NPs	0.05±0.001	0.04±0.008	0.05±0.006	0.06±0.006	0.05±0.001	0.05±0.001	0.04±0.008
BG-11 + 75 mg L^{-1} ZnO NPs	0.05±0.002	0.04±0.007	0.06±0.007	0.05±0.001	0.06±0.005	0.06±0.007	0.05±0.001
BG-11 + 0.75 mg L^{-1} ZnO NPs + 2 g L^{-1} NaCl	0.06±0.008	0.05±0.002	0.06±0.005	0.06±0.008	0.04±0.007	0.06±0.007	0.05±0.002
BG-11 + 7.5 mg L^{-1} ZnO NPs + 2 g L^{-1} NaCl	0.04±0.009	0.05±0.002	0.04±0.008	0.06±0.007	0.06±0.004	0.06±0.002	0.04±0.006
BG-11 + 75 mg L^{-1} ZnO NPs + 2 g L^{-1} NaCl	0.05±0.003	0.04±0.008	0.05±0.004	0.05±0.007	0.04±0.005	0.06±0.007	0.06±0.007
BG-11 + 0.75 mg L^{-1} ZnO NPs + 4 g L^{-1} NaCl	0.05±0.002	0.05±0.001	0.05±0.002	0.05±0.006	0.05±0.002	0.06±0.007	0.04±0.009
BG-11 + 7.5 mg L^{-1} ZnO NPs + 4 g L^{-1} NaCl	0.04±0.008	0.05±0.001	0.04±0.007	0.05±0.001	0.04±0.008	0.06±0.006	0.04±0.006
BG-11 + 75 mg L^{-1} ZnO NPs + 4 g L^{-1} NaCl	0.06±0.009	0.04±0.009	0.06±0.006	0.04±0.005	0.04±0.009	0.05±0.004	0.04±0.008

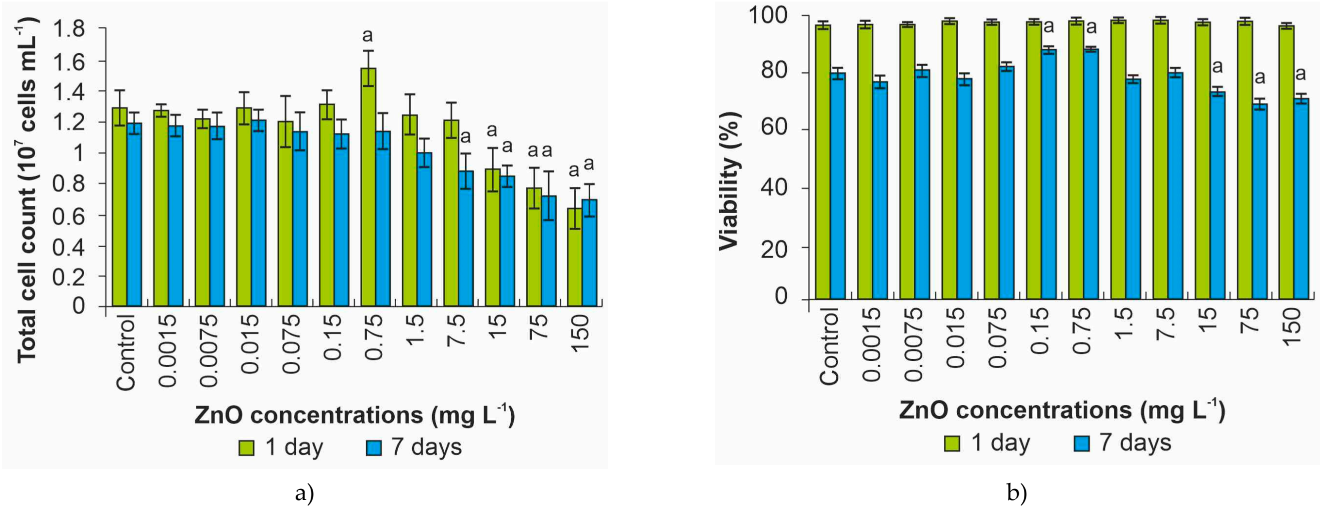

Figure S1. Effect of ZnO NPs on *Lobosphaera*: (**a**) total cell number; (**b**) viability. a - differences with control.
